# Advancing understanding of oat phenology for crop adaptation

**DOI:** 10.3389/fpls.2022.955623

**Published:** 2022-10-14

**Authors:** Ben Trevaskis, Felicity A. J. Harris, William D. Bovill, Allan R. Rattey, Kelvin H. P. Khoo, Scott A. Boden, Jessica Hyles

**Affiliations:** ^1^ Commonwealth Scientific and Industrial Research Organisation, Agriculture and Food Business Unit, Black Mountain Science and Innovation Park, Canberra, ACT, Australia; ^2^ Department of Primary Industries, Pine Gully Road, Wagga Wagga Agricultural Institute, Wagga Wagga, NSW, Australia; ^3^ School of Agricultural, Environmental and Veterinary Sciences, Charles Sturt University, Wagga Wagga, NSW, Australia; ^4^ Intergrain, Perth, WA, Australia; ^5^ School of Agriculture, Food & Wine, Faculty of Sciences, Waite Research Institute, University of Adelaide, Urrbrae, Adelaide, SA, Australia

**Keywords:** oat, flowering, seasons, vernalization, photoperiod

## Abstract

Oat *(Avena sativa)* is an annual cereal grown for forage, fodder and grain. Seasonal flowering behaviour, or phenology, is a key contributor to the success of oat as a crop. As a species, oat is a vernalization-responsive long-day plant that flowers after winter as days lengthen in spring. Variation in both vernalization and daylength requirements broadens adaptation of oat and has been used to breed modern cultivars with seasonal flowering behaviours suited to different regions, sowing dates and farming practices. This review examines the importance of variation in oat phenology for crop adaptation. Strategies to advance understanding of the genetic basis of oat phenology are then outlined. These include the potential to transfer knowledge from related temperate cereals, particularly wheat (*Triticum aestivum*) and barley (*Hordeum vulgare*), to provide insights into the potential molecular basis of variation in oat phenology. Approaches that use emerging genomic resources to directly investigate the molecular basis of oat phenology are also described, including application of high-resolution genome-wide diversity surveys to map genes linked to variation in flowering behaviour. The need to resolve the contribution of individual phenology genes to crop performance by developing oat genetic resources, such as near-isogenic lines, is emphasised. Finally, ways that deeper knowledge of oat phenology can be applied to breed improved varieties and to inform on-farm decision-making are outlined.

## Oat

Oat (*Avena sativa*) was potentially a wild food for Paleolithic hunter-gatherers (Lippi et al., 2015), before entering agriculture as a weed of wheat and barley during the Neolithic period (Murphy and Hoffman, 1992). As a weed, oat can dominate fields of wheat or barley and by 2000 years ago it was itself being grown as a crop (Harlan, 1982; Murphy and Hoffman, 1992). Factors that drove the acceptance of oat into early European farming systems included the ability to perform well in colder climates and marginal areas, with less inputs, together with end-use versatility that contributed to overall farm resilience (Moore-Colyer, 1995). The nutritional quality of oat grains, or groats, was also a key factor. Oat grains have high lipid content, contain lysine-rich protein and soluble fibre, particularly β-glucan (Cuddeford, 1995; Zwer, 2017). Oat grains are an ideal feed for horses, so there was strong demand for oats throughout the period when horses were a major contributor to transport and industry (Murphy and Hoffman, 1992). The same grain quality parameters are now driving interest in oats as a healthy food and as a non-animal protein source.

## The importance of phenology to oat adaption

Oat is an important crop in Australia, Canada, China, Europe, North and South America. Variation in the seasonal timing of life cycle events (phenology), particularly the timing of flowering and grain production, enables adjustment of the oat life cycle to suit local constraints. This variation is critical for the cultivation of modern oats across such a broad geographical range. For example, cultivation of oat in Canada is typically limited to spring and summer, thereby avoiding harsh winters. This contrasts with regions such as the United Kingdom where crops can be sown in autumn, then over-winter before producing grain in spring and summer. Other climate factors that can define the timing and duration of the growing season for oat include seasonal rainfall patterns and extreme summer heat.

The history of the Australian grains industry provides an example of the importance of variation in phenology for geographical adaptation. Oat came to Australia with Europeans and was cultivated in the Sydney area as early as 1791 (Collins and King, 1798). It was then slow to expand as a crop due to a lack of varieties suited to Australian growing conditions. This was because the oat growing season in south-eastern Australia is constrained by the timing of autumn rains, which dictate sowing dates, combined with the need to flower in the “optimal flowering period” to avoid frost, heat and water-limitation disrupting flowering and grain development (Tashiro and Wardlaw, 1990; Dolferus et al., 2011; Flohr et al., 2017). The European oats first introduced to Australia were ill-suited to the local growing conditions because they were slow to mature and flower. This remained the case until the early 20^th^ century when John Pridham, a cereal breeder trained by the pioneer wheat breeder William Farrer, began to breed locally adapted oat cultivars (Mengersen, 1960; Fitzsimmons, 1988). Pridham selected oats with earlier flowering, with his most successful variety being Belar (Fitzsimmons, 1988). Breeding of oats for Australian growing conditions continued with Pridham’s successors and this facilitated the expansion of the Australian oat industry (Mengersen, 1960). Now, on average, 1.4 million tons of milling oats are produced annually, with Western Australia and New South Wales being the major growing regions (ABARES, 2021). Additionally, around 0.7 million tons of oaten hay are exported annually, and more is used domestically as fodder (ABARES, 2021). Oat is also grown as a forage crop across large areas of New South Wales and Queensland.

Similar examples of how phenology adapts oat to local seasonal constraints can be found for other regions globally, though the specific of constraints differ to those in Australia (harsh winters in Canadian prairies, for example). In addition, as outlined in subsequent sections, phenology influences yield component traits, such as grain number, *via* determining pattens of plant growth and development, and so influences crop yield potential. The overall relationship between phenology and crop yield, through adaptation and yield potential, is not limited to Australian context. For example, phenology is a driver of yield in North American and European climates (Yan et al., 2007; Howarth et al., 2021).

From the perspective of overall farming systems, phenology also allows adaption to specific farm management practices. For example, selection for earlier flowering can allow double cropping in some farming systems (Islam et al., 2010). There is also evidence that in some regions breeders have selected for earlier maturity during the transition to mechanisation, which allowed sowing of large areas to occur within a narrower seasonal window (Grau Nersting et al., 2006). Similarly, phenology is an important component of optimisation of oat for different end uses. For example, the timing of maturation influences quality of milling oats (Howarth et al., 2021). Interactions between phenology and seasonal conditions also influence forage oat quality (Coblentz et al., 2012; Ki-Seung et al., 2014).

Phenology is likely to be of renewed global importance in an era of warming climates and increased climate variability. Re-optimisation of phenology might be required in some production zones. This could include adjustment of phenology to suit shifting sowing or harvest dates, and/or adjustment of phenology to compensate for more rapid growth and development driven by faster accumulation of growing degree days (Hunt et al., 2019; Peltonen-Sainto and Juahianen, 2020).

In summary, phenology enables adaption of oat to different climates, is a major determinant of crop yields, and can enable breeding of cultivars for different farming systems or end-uses. Subsequent sections review current understanding of oat phenology.

## Seasonal flowering behaviour of oat

**Figure 1:**
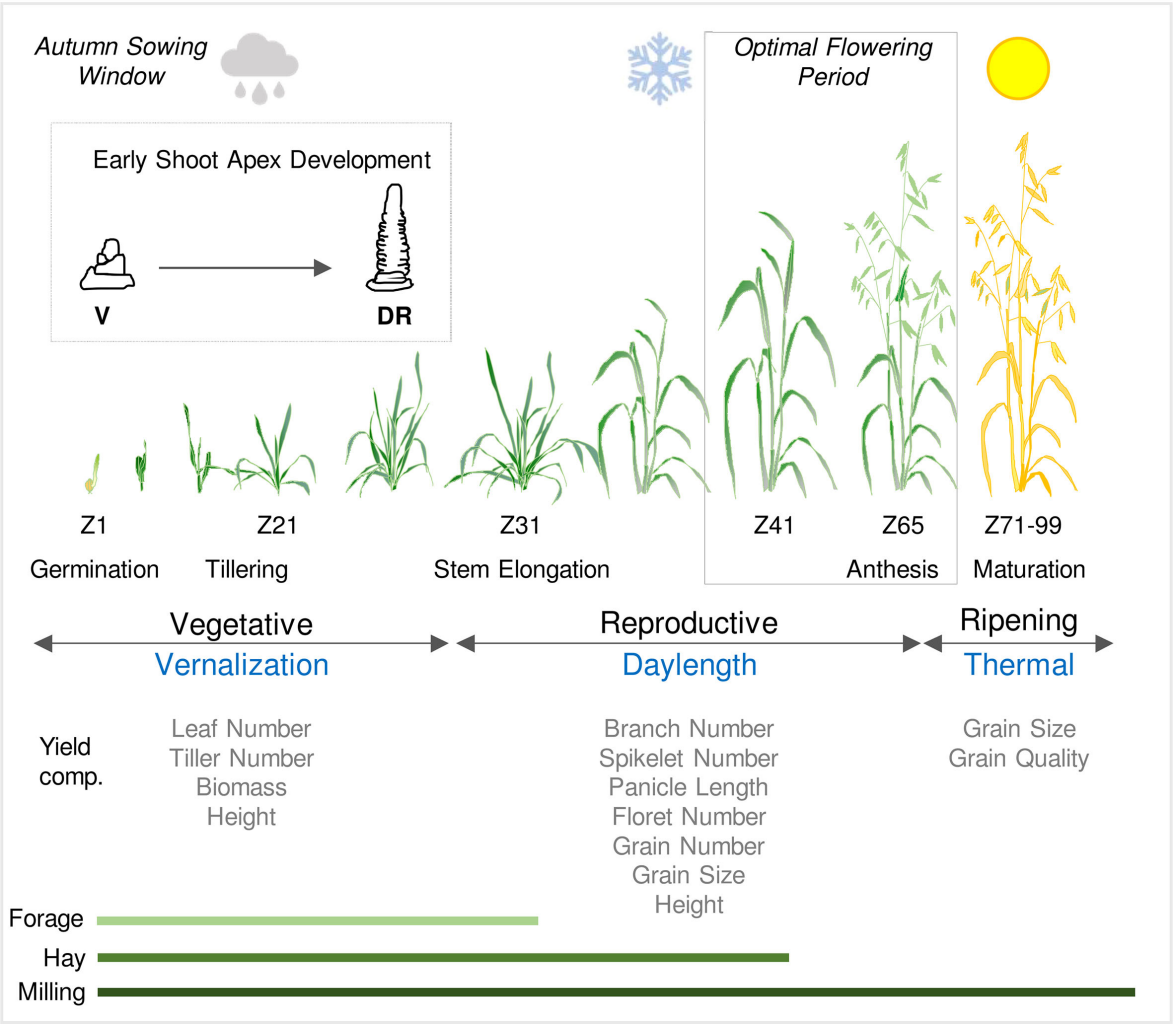
Schematic representation of the life cycle of oat in an Australian context. Sowing and establishment of crops is timed according to water availability in autumn (cloud symbol) and then plants experience cold temperatures through late autumn and winter (vernalization). Flowering and grain production occur during a optimal flowering period in spring, after the risk of frost subsides (snowflake symbol) and before the onset of heat and water limitation (sun symbol). Developmental stages are referenced by Zadoks scale (Zadoks et al., 1974). Inset shows early shoot apex development and the first visible sign of the transition from vegetative (V) to reproductive development; the double ridge stage (DR). The relationships between phases of development (vegetative and reproductive) and ripening are shown relative to the seasonal climate cues that influence the duration of each these phases. The impact of phase durations on physiological and yield related traits are indicated together with the relevance of growth phases to different end uses (forage, hay or milling).

The time of year when oat flowers and produces grain is determined largely by genotype-dependent responses to seasonal temperature and daylength (photoperiod) cues. As a species, cultivated oat can be described as a “vernalization-responsive long-day plant”, which will flower after exposure to winter cold (i.e., vernalization) as days lengthen in spring (**Figure 1**) (Borodin, 1934; Wiggans and Frey, 1955). This archetypal combination of seasonal flowering-responses is found in “winter” oats, which are well-suited to situations where crops are sown autumn and then overwinter before flowering in spring (Sampson and Burrows, 1972; Sorrells and Simmons, 1992). There is variation in both vernalization and daylength requirements amongst oat accessions/cultivars (Sampson and Burrows, 1972: King and Bacon, 1992). This variation has been harnessed by plant breeders to selectively modify oat phenology to suit diverse environments and different management practices, as outlined above.

Winter oats can require up to 50 days at low temperatures (below 10°C) to flower rapidly (Sorrells and Simmons, 1992). The response to cold is quantitative, such that increasing the duration of vernalization typically accelerates flowering to greater extents until the vernalization response is saturated (Sampson and Burrows, 1972). Vernalization accelerates the transition to flowering, the point when the shoot apex switches from vegetative to reproductive development (Bell, 1936; Bonnett, 1966; Aitken, 1974). A clear developmental indicator of the vernalization response, aside from earlier flowering, is reduction in the number of leaves produced on the main stem (final leaf number), since a vernalized plant spends less time in the vegetative phase, generating fewer phytomers and less leaves over the course of the plant life cycle (Aitken, 1974). A saturating vernalization treatment is one that generates the minimum final leaf number (Aitken, 1974). Whereas winter oats require vernalization to flower, spring oats generally show reduced or no requirement for vernalization (Borodin, 1934; Sampson and Burrows, 1972: King and Bacon, 1992). This allows spring oats to be sown when vernalization might not occur, after winter or in regions where winters are mild.

Daylengths exceeding 12 hours (long days) typically accelerate flowering of oat. The extent to which flowering is accelerated increases with longer daylengths, with the maximal response occurring when daylength exceeds 18 hours (Sampson and Burrows, 1972; Sorrells and Simmons, 1992). Daylength can influence the duration of the vegetative growth phase but, under typical field conditions, increasing daylengths during spring typically coincide with reproductive development and so have more impact on the duration of inflorescence development (Jenkins, 1973; Sorrells and Simmons, 1992) (**Figure 1**). While flowering of oat is usually delayed when plants are grown in daylengths shorter than 12 hours, some varieties flower rapidly irrespective of daylength (Burrows, 1984). There are examples of oats that flower rapidly irrespective of both vernalization and daylength (Aitken, 1974).

## Cereal phenology genes

For the reasons outlined above, selecting optimal phenology is a key goal for oat breeding programs, so understanding the genetic basis for variation in oat phenology can contribute to future breeding strategies. The molecular basis of variation in oat phenology has not been resolved. There is, however, detailed knowledge of the gene sequences that underlie variation in the vernalization and daylength requirements of other temperate cereals, particularly wheat and barley (see Fjellheim et al., 2014; Hyles et al., 2020). The *Avena* genus is part of the same subfamily of grasses as wheat and barley (Pooideaea) and all are members of the “Core Pooid” clade. This close evolutionary relationship, combined with the similar flowering physiology, suggests that there are good prospects to transfer knowledge of the molecular basis of seasonal flowering from wheat and barley to oat.

## Vernalization

The key gene controlling vernalization-induced flowering of wheat and barley is the MADS box transcription factor, *VERNALIZATION1* (*VRN1*) (Danyluk et al., 2003; Trevaskis et al., 2003; Yan et al., 2003). *VRN1* promotes the transition to reproductive development but is transcribed at low levels in plants that have not been vernalized. Exposing plants to prolonged cold activates transcription of *VRN1* and this subsequently accelerates flowering when plants are grown at warmer temperatures (Danyluk et al., 2003; Trevaskis et al., 2003; Yan et al., 2003; Chen and Dubcovsky, 2012). Activation of *VRN1* by cold is quantitative such that *VRN1* transcript levels increase more with longer cold treatments, accelerating flowering to greater extents (Danyluk et al., 2003; Trevaskis et al., 2003; Yan et al., 2003; von Zitzewitz et al., 2005; Sasani et al., 2009). Spring wheats that flower without vernalization typically carry alleles of *VRN1* that are transcribed without cold and so bypass the requirement for vernalization (Pugsley, 1971; Yan et al., 2003; Yan et al., 2004a; Fu et al., 2005; Eagles et al., 2009; Eagles et al., 2011; Díaz et al., 2012; Oliver et al., 2013). These alleles have mutations in the promoter or first intron that are suggested to trigger elevated transcriptional activity (Yan et al., 2003; Yan et al., 2004a). *VRN1* copy number variation and amino acid substitutions have also been linked to variation in vernalization requirement of wheat (Chen et al., 2009; Díaz et al., 2012; Dixon et al., 2019).

## Daylength

The mechanisms underlying the daylength flowering response were first resolved in *Arabidopsis thaliana* (Arabidopsis) where acceleration of flowering by long days is mediated by *FLOWERING LOCUS T* (*FT*) (Kardailsky et al., 1999; Kobayashi et al., 1999; Corbesier et al., 2007). *FT* encodes a small protein, often described as a florigen, that is expressed in leaves in long days and then transported to the shoot apex where it triggers flowering (Corbesier et al., 2007; Tamaki et al., 2007). Activation of *FT* expression in leaves is activated by a molecular network that includes phytochromes, which perceive external light cues, and the circadian oscillator, which gives rise to rhythmic day-night gene expression patterns that mediate daylength (or photoperiod) responses (Suarez-Lopez et al., 2001; Valverde et al., 2004). The photoperiod response pathway first elucidated in Arabidopsis seems to be widely conserved in plants, including wheat and barley, where transcription of a *FT*-like gene (*FT1*, also known as *VERNALIZATION3, VRN3*) is activated in the leaves by long days (Turner et al., 2005; Yan et al., 2006).

**Figure 2:**
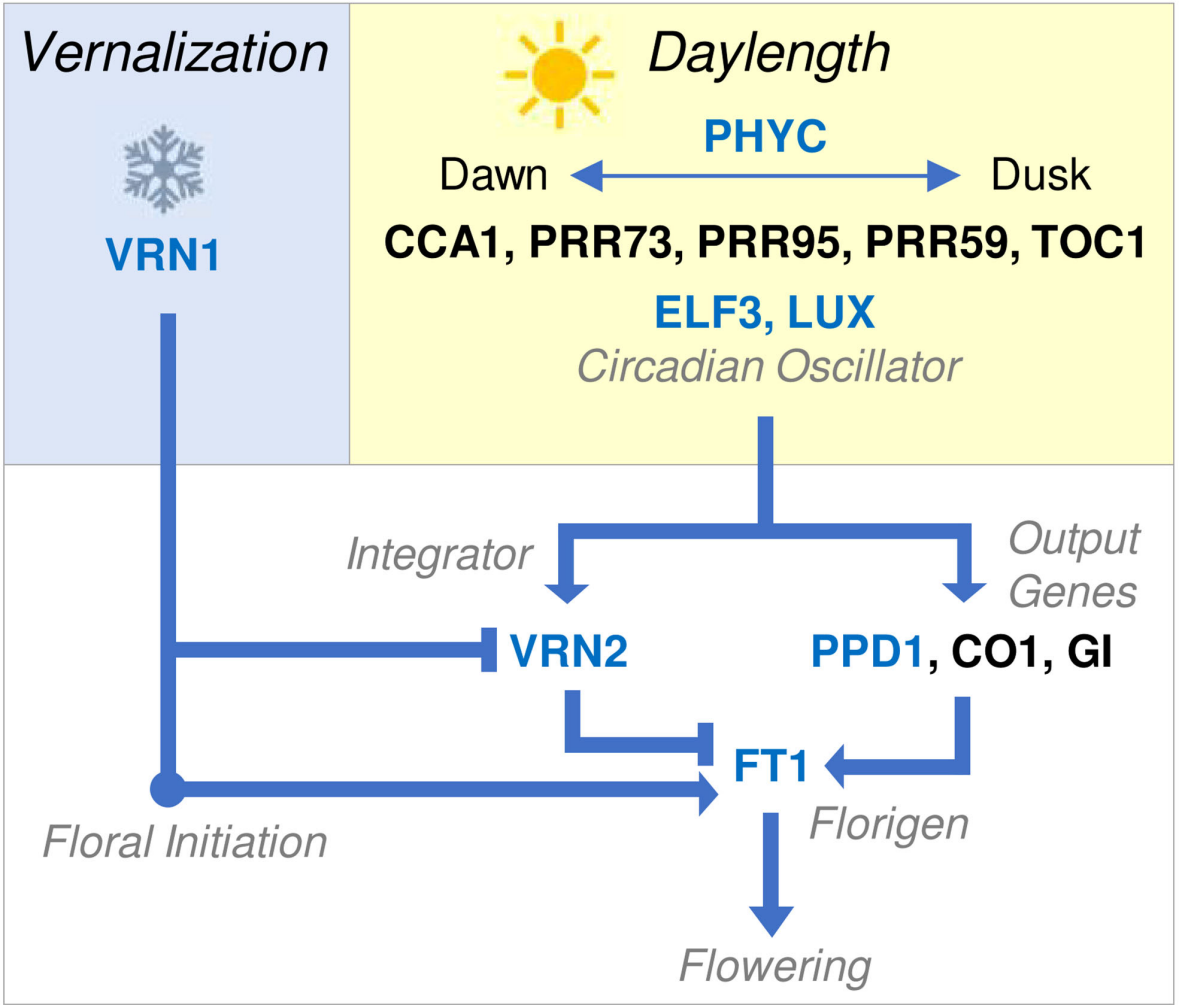
Overview of molecular genetic network controlling the seasonal flowering behaviour of temperate cereals. Vernalization (blue box) activates *VRN1*. This is sufficient to trigger the transition to reproductive development or floral initiation, as indicated by the double ridge stage of shoot apex development. Additionally, *VRN1* represses *VRN2* and activates *FT1*, allowing the daylength flowering response to occur after vernalization. Daylength perception (yellow box) is mediated by the circadian oscillator, which generates internal biological rhythms that allow perception of external photoperiod. The circadian oscillator regulates output genes (white box) that activate *FT1*, the key trigger for daylength-induced flowering also known as florigen. *VRN2* is also daylength responsive and blocks long-day induction of *FT1*, until *VRN2* is itself repressed by *VRN1*. *FT1* accelerates reproductive development and stem elongation, leading to flowering. Arrows indicate activation of a target gene, lines ending in bars indicate repression. Blue text indicates that a gene has been linked to (or associated with) natural variation for seasonal flowering behaviour. Gene names are shown without italics for clarity and blue text indicates genes known to mediate natural variation in phenology of other cereals.

Mutations in genes that mediate circadian oscillator function and output have been linked to variation for daylength sensitivity. For example, the main gene that determines daylength sensitivity of wheat and barley is *PHOTOPERIOD1 (PPD1)* (Turner et al., 2005). *PPD1* is related to *PSEUDORESPONSE REGULATOR* genes that contribute to circadian oscillator function (Turner et al., 2005; Beales et al., 2007). In photoperiod sensitive wheats, which require long days to flower, *PPD1* is expressed with an oscillating expression pattern through day-night cycles and mediates activation of *FT* expression when days are long or increasing in length (i.e., spring and summer) (Beales et al., 2007). Mutations that alter *PPD1* activity are a common basis for variation in the photoperiod sensitivity of wheat and barley. For example, a deletion in the promoter of the D genome of *PPD1* is associated with high *PPD1* expression throughout day-night cycles and, in turn, with elevated *FT1* expression and earlier flowering in short days (Beales et al., 2007; Gauley and Boden, 2021). Conversely, coding sequence mutations in the barley *PPD1* gene that likely inhibit function of the PPD1 protein are linked to delayed flowering under long days (Turner et al., 2005). Copy number variation for the *PPD1* gene has also been linked to variation in photoperiod sensitivity of wheat (Díaz et al., 2012). Other genes that function in the circadian oscillator influence phenology of wheat and barley, including *LUX/ARRYTHMO* and *EARLY FLOWERING 3* (Mizuno et al., 2012; Faure et al., 2012; Zakhrabekova et al., 2012; Campoli et al., 2013; Boden et al., 2014; Gawroński et al., 2014; Zikhali et al., 2016). Additionally, *PHYTOCHROME C (PHYC)* plays a major role in daylength perception in cereals and can influence the photoperiod flowering response (Chen et al., 2014). Similar to *PPD1*, all of these genes seem to influence flowering behaviour, at least in part, by influencing *FT1* expression (**Figure 2**).

## Integration of vernalization and daylength pathways

Vernalization is normally a pre-requisite for long-day induction of *FT1* (Hemming et al., 2008). This is mediated by the *VERNALIZATION2 (VRN2)* gene, which limits expression of *FT1* in long days to repress flowering prior to vernalization (Yan et al., 2004b; Trevaskis et al., 2007; Hemming et al., 2008). Loss of *VRN2* function (e.g., through gene deletion) allows long-day induction of *FT1* expression without plants experiencing prolonged cold and thereby bypasses the vernalization requirement (Yan et al., 2004b; Hemming et al., 2008). This occurs mainly in barley and diploid einkorn wheat but is unlikely to be common in tetraploid durum or hexaploid bread wheats, where gene redundancy reduces the likelihood of complete loss of *VRN2* function. Alleles of *FT1* that are expressed at high basal levels can bypass *VRN2* and trigger rapid flowering irrespective of vernalization status or daylength (Yan et al., 2006). The precise genetic basis for these alleles has not been resolved; copy number, local rearrangements and insertions/deletions have all been associated with ‘early’ alleles of *FT1* (Yan et al., 2006; Loscos et al., 2014; Nitcher et al., 2014).

## The relationship between phenology genes, development and yield component traits of cereals

Phenology influences other aspects of oat biology in addition to determining when flowering and grain production occur. Vernalization can alter tiller number and there is a strong relationship between the duration of vegetative growth phase and the capacity to survive freezing winter temperatures, as is the case for other temperate cereals (Sorrells and Simmons, 1992; Wooten et al., 2009). By influencing the duration of inflorescence development, the daylength flowering response influences inflorescence structure, such that longer daylengths reduce the duration of inflorescence development and decrease the number of spikelets produced by the oat panicle (Finkner et al., 1973; Peltonen-Sainto, 1994). Genes that determine the duration of vegetative or reproductive development can influence inflorescence node and spikelet number of wheat, barley and rice (*Oryza sativa*). Examples include the *PPD1* and *FT1* genes of wheat and also the *HEADING DATE1* and *EARLY HEADING DATE 1* genes of rice (Endo-Higashi and Izawa, 2011; Boden et al., 2015; Dixon et al., 2018; Finnegan et al., 2018; Brassac et al., 2021). The common theme from these examples is that genes that reduce the duration of reproductive development can decrease the number of inflorescence nodes produced and thus the number of spikelets and florets produced by each plant. This can in turn impact the total grain number per unit area and thereby influence yield. This parallels observations that vernalization and daylength influence tiller or spikelet number of oat (see above). So, in addition to playing a central role in adaptation, oat phenology genes are likely to influence other traits that underpin grain yield, as is the case for other temperate cereals and rice (see Trevaskis, 2018) (**Figure 1**).

## Genes that drive variation in oat phenology

A role for *VRN1*-like genes in mediating vernalization-induced flowering appears to be broadly conserved in Pooid grasses (Ream et al., 2014; McKeown et al., 2016; Feng et al., 2017; Woods et al., 2017). This suggests that *VRN1*-like genes are likely to play a role in vernalization-induced flowering of oat, a member of the core Pooid grasses. Direct support for this idea comes from observations that an oat *VRN1* orthologue is induced by vernalization in winter cultivars but is expressed at high levels irrespective of vernalization status in spring cultivars (Preston and Kellogg, 2008). Additionally, analysis of *FT1* expression patterns across the Pooid clade suggests that long-day induction of *FT1* was also a feature of the common ancestor of this group of grasses (Ream et al., 2014; McKeown et al., 2016). If the molecular networks controlling the seasonal flowering behaviour of oat are similar to those of wheat and barley, a key question then becomes; does variation for vernalization requirement or photoperiod sensitivity map to oat homologues of *VRN1* or *FT*?

Several studies describe genetic mapping of oat loci that contribute to variation in the timing of flowering (Wight et al., 1994; Holland et al., 1997; Holland et al., 2002; Portyanko et al., 2005; Locatelli et al., 2006; Wooten et al., 2009; Nava et al., 2012; Herrmann et al., 2014; Esvelt Klos et al., 2016; Sunstrum et al., 2019). These studies used a range of mapping populations, including recombinant inbred lines and diversity panels, which were genotyped using different marker systems. Phenotyping was conducted in diverse conditions including controlled environments, with different vernalization or daylength treatments, and at a range of field locations with different sowing dates across different years. Many of these studies linked phenological variation to chromosomal locations where *VRN1* or *FT1* are likely to be located according to cross-species comparisons of genetic maps. For example, Holland et al. (2002) suggested the marker BCD808b, which is linked to variation in vernalization responsiveness, is located near a copy of the *VRN1* gene. Another study amplified *VRN1* and *FT1* gene sequences directly from oat, identified nucleotide polymorphisms in these sequences, and then used these to assign potential locations for these genes on genetic maps (Nava et al., 2012). This study also concluded that *VRN1* is linked to variation in oat phenology.

The assembly of hexaploid (AACCDD) oat reference genome sequences (Maughan et al., 2019; Kamal et al., 2022; Peng et al., 2022) now allows direct comparisons between the physical position of candidate phenology genes and the locations of genetic markers linked to variation in flowering behaviour. These comparisons support the idea that *VRN1* and *FT1* are likely to underlie variation in oat phenology, with *VRN1* homeoalleles on chromosomes 4D, 7A and 7D, and *FT1* homeoallele on 7A all linked to phenological variation (Tinker et al., 2022). *FT1* is also potentially linked to the *DAYLENGTH INSENSITIVITY1 (Di1)* gene, on chromosome 7D, which reduces daylength sensitivity by accelerating flowering in short days and has been used to breed rapid cycling oats for Canada and Brazil (Burrows, 1984; Locatelli et al., 2006; Tinker et al., 2022).

It should be noted that there are other candidate genes within the chromosomal regions of interest outlined above. For example, *PHYC* and an oat homologue of *HEADING DATE* 6 *(HD6)* (Takahashi et al., 2001) are co-located with *VRN1*. This is consistent with the suggestion of Sunstrum et al. (2019) that the genetic interval where *VRN1* is located might combine effects from multiple genes, including genes that influence flowering in a vernalization-independent manner. Alternative candidate genes have also been suggested for the region linked to *Di1*, including a *CONSTANS*-like gene. (Chaffin et al., 2016; Sunstrum et al., 2019; Tinker et al., 2022). This region is difficult to resolve because marker-trait relationships are inconsistent across populations and there is also evidence for reduced recombination rates (Chaffin et al., 2016; Sunstrum et al., 2019; Tinker et al., 2022). Nevertheless, *VRN1* and *FT1* genes are promising candidates for more detailed analyses, while *PHYC* and *HD6* also warrant further characterisation.

In summary, variation in oat phenology has been mapped to chromosomal regions containing genes related to those that control vernalization and daylength sensitivity in other cereals. Detailed understanding into the nature of the variation in these genes, and how this affects gene function and overall flowering behaviour, is not yet available.

## Advancing understanding of oat phenology for adaptation to Australian farming systems

A deeper understanding of the role that oat phenology genes play in adaptation can contribute to the future success of Australian oats through breeding and by contributing to on farm management decision-making. Additionally, there is potential to use phenology genes to optimise plant architecture and thereby increase crop yield.

A first step towards developing an increased understanding of the genetic basis of oat phenology is to identify “major genes” that control vernalization requirement and photoperiod sensitivity. Current knowledge allows the genetic intervals containing these genes to be identified (see above). Future studies will focus on validating and further characterising “causal genes”, as has been achieved in wheat and barley. A number of high-resolution mapping approaches can be used to achieve this aim, but ultimately a test of gene function, such as mutagenesis or gene editing, will be required to verify candidate gene functions (e.g., Boden et al., 2015). Additionally, as has been the case in wheat and barley, detailed analysis of gene expression patterns in diverse oat varieties, in response to different vernalization or photoperiod treatments, can provide further insights into the potential roles of candidate phenology genes.

Already there is scope for surveys of genetic diversity using high-throughput sequencing, either of whole genomes or by targeting candidate genes in the regions already identified by genetic mapping and cross-species comparisons. Sequencing of oat pangenomes (https://wheat.pw.usda.gov/GG3/PanOat) will further accelerate this approach. The collected pedigrees of oat varieties, which have been recorded and curated by breeders over many decades (Tinker and Deyl, 2005), are a valuable resource for surveying genetic diversity of major phenology genes. Access to pedigrees allows diversity surveys to be targeted to accessions and cultivars that represent key lines from the history of oat breeding, including key founders and breeding parents spanning the entire period from development of early modern varieties to current elite cultivars (see Eagles et al., 2009). Understanding the genetic basis of phenology and adaptation of oats that were bred for different regions is a logical approach to understand adaptation to local climate and farming practices.

**Figure 3:**
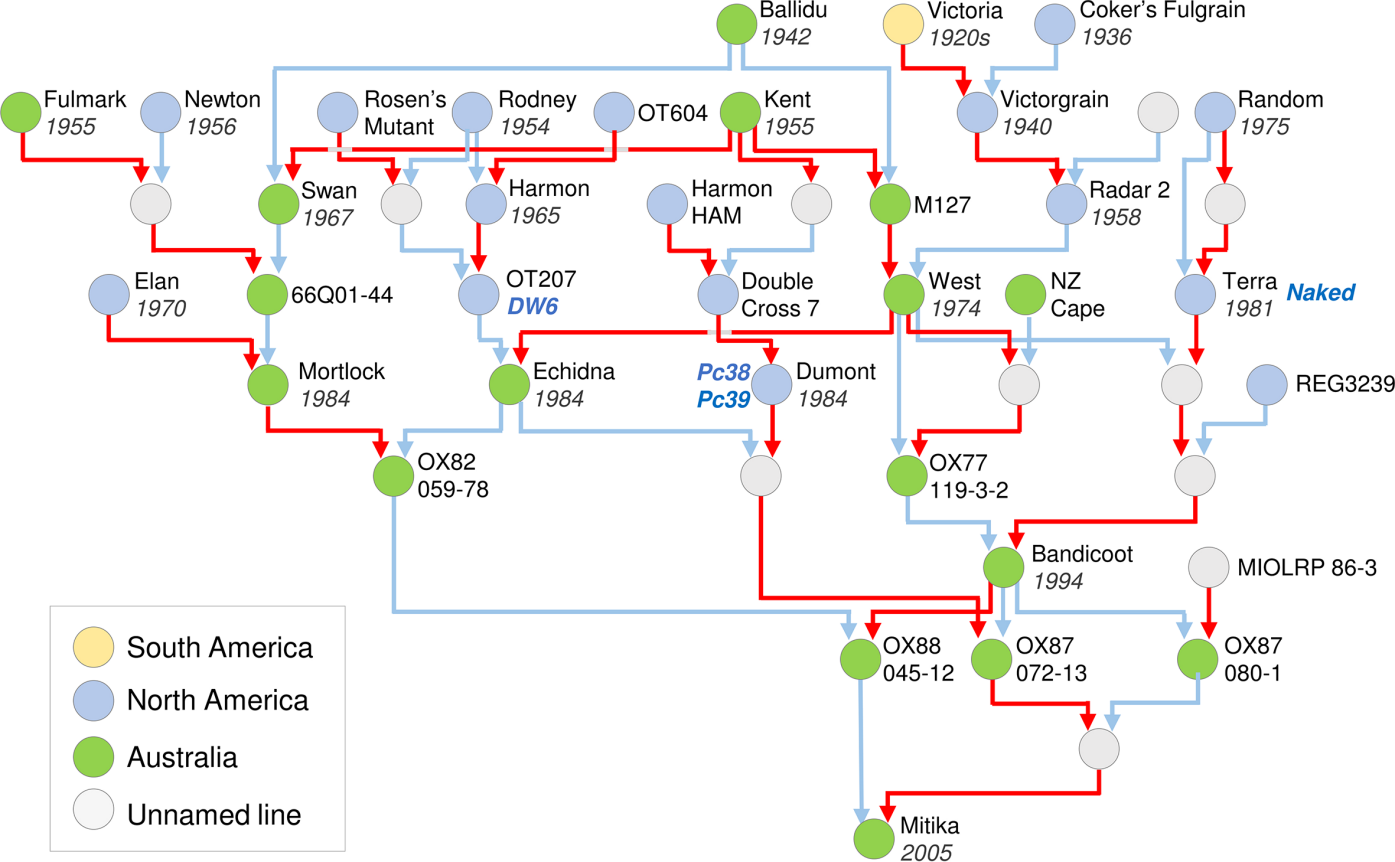
Pedigree of an elite Australian spring oat cultivar, cv. Mitika. A simplified pedigree of cultivar Mitika, modified from a Helium output (Shaw et al., 2014) generated from the pedigrees of over 1000 international accessions spanning diversity relevant to the history of Australian oat breeding. Pedigrees are shown as far back as early 20^th^ century oats from Australian and North America. For ease of representation, multiple rounds of backcrossing are not shown. Pedigrees were obtained from the “Pedigrees of Oat Lines” POOL database (Tinker and Deyl, 2005), from Fitzsimmons et al. (1983) and directly from oat breeders (Dr Pamela Zwer and Dr Bruce Winter, personal communication). The year of release is indicated for cultivars. Red line indicates maternal parent connection and blue indicates paternal parent (pollen donor). Colours of circles indicate region of origin for released lines. Grey circles indicate unnamed intermediate lines used in crossing.

An example of an oat pedigree is presented for Mitika, a South Australian milling oat that was registered in 2003 (**Figure 3**). This example highlights how pedigrees can be used to identify the donors of key traits into a local breeding program, including breeding line OT207 for the *DWARF6 (DW6)* reduced height gene, the Canadian cultivars Terra for the *NAKED1* gene for hull-less grain and Dumont for crown rust (*Puccinia coronata*) resistance genes *Pc38* and *Pc39* (Brown et al., 1980; McKenzie et al., 1984; Ubert et al., 2017). The pedigree of Mitika also shows extensive mixing of Australian and North American germplasm. Also noteworthy is that all ancestors shown are described as spring types. This suggests that there is potential to introduce more variation for vernalization requirement into Australian milling oats to suit specific environments or farming systems opportunities, such as earlier sowing and dual-purpose crops (graze and grain).

Knowledge of diversity in the major phenology genes can be used in breeding to facilitate choice of parents with compatible phenology for crossing and/or to select progeny of crosses, using molecular markers. Based on knowledge of wheat and barley there is potential for multiple functional alleles of major phenology genes (i.e., many variants of each gene) that have different impacts on phenology and/or yield (Eagles et al., 2009; Hemming et al., 2009; Eagles et al., 2010; Eagles et al., 2011; Díaz et al., 2012). Molecular markers should ideally have the capacity to resolve different functional haplotypes or alleles (Eagles et al., 2010; Eagles et al., 2011). Design of such markers requires high-resolution assays of genetic variation at key loci, using gene resequencing or high-density genome-wide Single Nucleotide Polymorphism (SNP) assays, for example. There then needs to be functional understanding of how different haplotypes influence phenology. Development of near-isogenic lines, by recurrent backcrossing, is a reliable way to contrast the effects of different alleles on phenology; different haplotypes identified by diversity surveys can be introgressed into a common genetic background for phenotyping in different controlled and field conditions (e.g., Hunt et al., 2019). Ideally a successful modern oat that performs well in target environments would be chosen as the recurrent parent that provides the genetic background for near-isogenic lines (e.g., Mitika or Bannister for Australia). Since oat is transformable there is also potential to develop and test novel variation in phenology using gene editing (Gasparis et al., 2008; Matres et al., 2021). Ideally this will target modern elite cultivars that are most relevant to the grains industry.

If the impact of different alleles of the major phenology genes can be resolved then a key question becomes; how can detailed knowledge of individual genes derived from reductionist approaches be re-integrated to understand, predict or optimise overall crop performance? This is a complex challenge that needs to consider both gene-gene and gene-environment interactions. There is also a “sparse data” challenge because detailed phenological observations will only ever be available for specific genotypes grown at particular field sites or sowing dates, representing only a subset of all the scenarios that potentially exist. One effective approach to address these challenges is to integrate genetic knowledge into physiology-based predictive models, such as Agricultural Production Systems Simulation (APSIM, Keating et al., 2003; Zheng et al., 2013). Predictive tools like APSIM can use genetic knowledge to help inform on-farm decision-making. For example, genotypes for vernalization and photoperiod genes can be incorporated into simulation models that predict the date of flowering for specific cultivars, from different sowing dates. Combined with climate data, these predictions can be used by farmers to select sowing dates for cultivars that minimise frost or heat risk. (Zheng et al., 2013).

**Figure 4:**
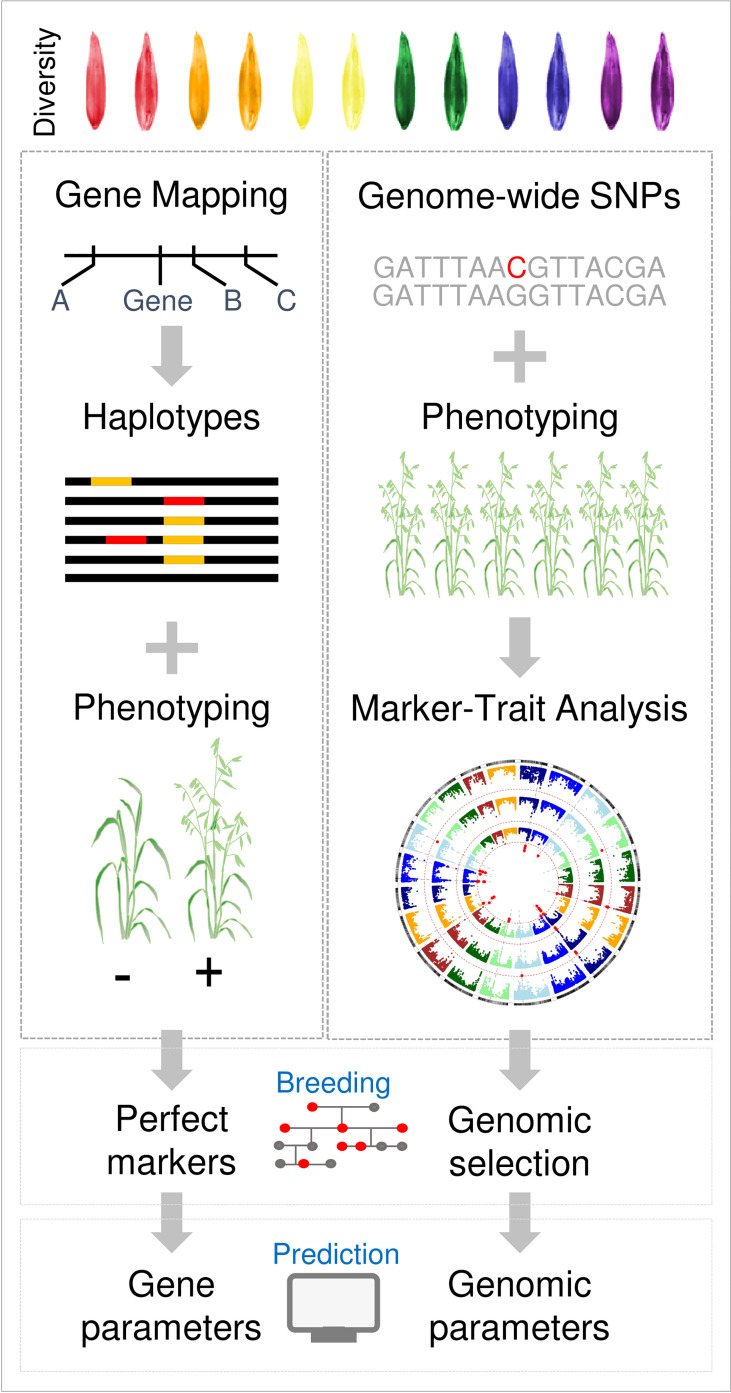
Overview of future oat phenology research strategies. A traditional strategy, based on approaches applied to wheat and barley, is compared with a “genome-to-phenome” strategy. In the traditional approach individual phenology traits are mapped to genes, using bi-parental mapping populations, for example. Knowledge of variation in these genes is then assembled by gene resequencing and used to design molecular markers for genotyping. In parallel, bespoke genetic resources such as near-isogenic lines or induced mutants are used to test gene function. In the genome-to-phenome strategy diversity panels are used to link genome-wide genetic diversity to broad phenotypic variation (phenology in this case). Marker-trait associations are then used for genomic prediction or to inform crop simulation models that predict phenology.

The research strategies outlined above were tried and tested in wheat and barley research over a period of more than two decades. Advances in genomics, phenomics and biological data-science allow an alternative “genome-to-phenome” approach to be applied to understand phenology and adaptation of oats (**Figure 4**). Core to this approach is to survey genetic diversity at high-resolution and genome-wide scale in a representative population that captures the flow of alleles through the history of oat breeding, spanning founders, international imports, key parents and modern elite cultivars. The advent of high-resolution oat genotyping-by-sequencing assays can facilitate this (Bekele et al., 2018; Bekele et al., 2020). Another approach is to generate population-scale transcriptomes from key organs or timepoints in the plant lifecycle. Transcriptome data can be used to detect SNP variation in the coding regions of genes that are likely to function in the relevant tissues/timepoints of interest and can also assay variation in gene expression states across the genome, in different accessions (Xu et al., 2015). By phenotyping the same panel under controlled conditions (long versus short days, with or without vernalization), together with industry relevant field trials, it is possible to link variation in phenology to chromosomal regions. This generates data at scale that can be used to resolve gene-by-environment interactions through the application of emerging analytical tools, such as machine learning. Access to marker-trait associations for phenology at genome-wide scale in controlled environments that resolve variation for discrete aspects of phenology (i.e., vernalization and daylength requirements) also allows genomic parameterisation of simulation models or other data-driven prediction methods. Specifically, marker-trait association data can be used to set vernalization and daylength response parameters in APSIM to predict the flowering behaviour of individual oat cultivars. The same marker-trait association data can then be utilised in crop breeding through genomic selection/prediction. A “genome-to-phenome” strategy could be used alongside a more traditional approach and has the advantage that the genomic resources developed can be used to explore other aspects of oat biology.

## Conclusions

Variation in phenology underlies adaptation of oat to climates, farming systems and end uses. Rapid progress in the development of genomic resourcess and the assembly of an oat reference genome sequence is accelerating progress towards understanding genes that underlie variation in phenology. Comparison of the physical locations of genetic markers linked to variation phenology with the likely locations of candidate phenology genes in the oat genome suggests that *VRN1* and *FT1* contribute to natural variation in oat phenology. There is now a need for detailed studies of the expression of these genes in diverse oat varieties, in response to different vernalization or photoperiod treatments, together with functional validation by mutagenesis or gene editing. Other future priorities will be to determine how haplotypes of phenology genes contribute to variation in oat phenology and to overall crop performance, by generating near-isogenic lines, for example. This knowledge can then be incorporated into genomic selection strategies, which can be used in crop breeding, and into phenology prediction tools that can be used to inform on-farm decision-making.

## Author contributions

All authors listed have made a substantial, direct, and intellectual contribution to the work and approved it for publication.

## Funding

This work was jointly funded by the Grains Research and Development Corporation and CSIRO, project code CSP2007.

## Acknowledgments

The authors wish to thank Dr Howard Eagles for his guidance and mentorship over several years. We also thank Dr Pamela Zwer and Dr Bruce Winter for providing pedigree information.

## Conflict of interest

The authors declare that this study received funding from the Grains Research and Development Corporation (GRDC). The funder was not involved in the study design, collection, analysis interpretation of data, the writing of this article or the decision to submit it for publication.

## Publisher’s note

All claims expressed in this article are solely those of the authors and do not necessarily represent those of their affiliated organizations, or those of the publisher, the editors and the reviewers. Any product that may be evaluated in this article, or claim that may be made by its manufacturer, is not guaranteed or endorsed by the publisher.
